# Angiographic Success Does Not Fully Reflect Tissue-Level Reperfusion: New Diffusion-Weighted Imaging Lesions After True Complete (TICI 3) Recanalization

**DOI:** 10.3390/diagnostics16091288

**Published:** 2026-04-25

**Authors:** Feyza Sönmez Topcu, Arsida Bajrami, Sena Aksoy, Songül Şenadım, Serdar Geyik

**Affiliations:** 1 Department of Radiology, Istanbul Aydin University, Medical Park Florya Hospital, 34295 Istanbul, Turkey; drserdarg@hotmail.com; 2 Department of Neurology, Istanbul Aydin University, Medical Park Florya Hospital, 34295 Istanbul, Turkey; arsidabajrami@gmail.com (A.B.); aksoysena13@gmail.com (S.A.); songulsenadim@gmail.com (S.Ş.)

**Keywords:** diffusion-weighted imaging, mechanical thrombectomy, magnetic resonance imaging, embolic stroke, reperfusion

## Abstract

**Background and Purpose:** Complete angiographic reperfusion (TICI 3) is considered the optimal procedural endpoint of mechanical thrombectomy (MT) in acute ischemic stroke. However, new diffusion-weighted imaging (DWI) lesions are frequently observed despite apparent angiographic success. We aimed to investigate the incidence, morphological patterns, and clinical relevance of these lesions in a strictly defined TICI 3 cohort. **Methods:** In this retrospective single-center study, 89 patients with anterior circulation large-vessel occlusion (LVO) who achieved true TICI 3 were analyzed. Baseline and follow-up Magnetic Resonance Imaging (MRI) within 48 h were systematically compared using paired diffusion-weighted imaging (DWI) and apparent diffusion coefficient (ADC) maps to identify new lesions. Lesions were classified according to morphology and distribution. Stroke etiology was assessed using TOAST criteria. Functional outcomes were evaluated using the 90-day modified Rankin Scale (mRS) with the Rankin Focused Assessment. **Results:** New DWI lesions were detected in 28 of 89 patients (31.5%). The predominant pattern was millimetric cortical foci (85.7%), most frequently ipsilateral to the recanalized vessel (78.6%), with fewer contralateral (14.3%) and bilateral (7.1%) lesions. Territorial infarcts and isolated basal ganglia infarcts were each identified in 14.3% of patients, with some overlap between categories. No significant differences were observed between patients with and without new lesions regarding baseline characteristics or procedural metrics (all *p* > 0.05). Importantly, the presence of new DWI lesions was not associated with 90-day functional outcome (*p* = 0.930) or survival (*p* = 0.613). **Conclusions:** New DWI lesions are common even after complete angiographic reperfusion, highlighting a persistent dissociation between macrovascular success and tissue-level integrity. Although predominantly small and clinically silent in the short term, these findings underscore the limitations of angiographic endpoints alone and support the need for strategies targeting microvascular protection and prevention of distal embolization.

## 1. Introduction

The therapeutic landscape of acute ischemic stroke has undergone a paradigm shift over the past decade. The publication of landmark randomized controlled trials in 2015 and 2016, including MR CLEAN, ESCAPE, EXTEND-IA, SWIFT PRIME, and REVASCAT, established mechanical thrombectomy (MT) as the standard of care for patients with anterior circulation large-vessel occlusion (LVO) [1,2,3,4,5]. These pivotal studies demonstrated that rapid endovascular recanalization improves functional independence compared with medical therapy alone, leading to a global reorganization of stroke care systems to prioritize timely access to neurointerventional procedures.

The primary objective of MT is maximal angiographic reperfusion, typically quantified using the Thrombolysis in Cerebral Infarction (TICI) scale. Complete reperfusion, defined as TICI grade 3, is widely regarded as the gold-standard procedural endpoint. Large-scale meta-analyses, including the HERMES collaboration, have shown that higher reperfusion grades correlate with better clinical outcomes, lower mortality, and smaller final infarct volumes [6,7,8]. Accordingly, technical refinements in stent-retriever and aspiration techniques have increasingly focused on achieving first-pass TICI 3 recanalization, which is associated with the highest probability of favorable recovery.

However, a clinic—radiological paradox is frequently encountered: a substantial proportion of patients do not achieve functional independence despite technically “perfect” angiographic results. This phenomenon—often termed futile reperfusion or incomplete microvascular recovery—suggests that large-artery patency does not always translate into effective tissue-level perfusion [9,10,11]. Even after removal of the primary thrombus, distal microembolization, no-reflow, and ischemia–reperfusion-related thrombo-inflammatory injury may contribute to ongoing tissue damage [10,12,13].

Follow-up neuroimaging, particularly diffusion-weighted imaging (DWI), plays a critical role in assessing tissue recovery after endovascular therapy. DWI is highly sensitive to early cytotoxic edema and can detect ischemic lesions within minutes. It is widely used in the acute and early post-treatment period to assess both initial infarct burden and subsequent lesion evolution. Imaging within the first 24–48 h is particularly valuable for identifying procedure-related ischemic changes while minimizing confounding effects of delayed infarct progression. Notably, new DWI-restricted lesions are often observed on post-procedural Magnetic Resonance Imaging (MRI) even in patients who achieve immediate TICI 3 reperfusions [14,15,16]. These lesions may occur within the target territory, in new vascular territories, or in the contralateral hemisphere. Reported incidences vary widely, reflecting heterogeneity in imaging timing, patient selection, and thrombectomy technique [13,17,18].

The pathophysiology of new post-procedural DWI lesions is likely multifactorial. A key mechanism is iatrogenic distal embolization during thrombus manipulation and device deployment or retrieval [18,19]. High-resolution MRI studies suggest that many lesions represent sub-angiographic peripheral emboli—fragments too small to be visualized on conventional Digital Substruction Angiography (DSA) yet sufficient to produce focal infarction [13]. In addition, no-reflow—microvascular obstruction driven by pericyte constriction, endothelial swelling, and leukocyte adhesion—may limit effective perfusion despite large-vessel patency and contribute to new or expanding DWI abnormalities [10].

Despite their frequency, the clinical relevance of these often millimetric lesions remains debated. Some argue that any new infarct reflects incomplete neuroprotection and may contribute to cognitive decline or subtle deficits [20,21,22]. Others suggest that after successful TICI 3 reperfusion, small lesions are frequently clinically silent and do not influence 90-day disability as measured by the modified Rankin Scale (mRS) [23,24]. This uncertainty is reflected in evolving definitions of “successful” reperfusion, with increasing emphasis on tissue-level endpoints alongside angiographic scores [25,26,27].

Importantly, the definition of “new” lesions varies across studies, particularly with regard to distinguishing true de novo ischemic foci from lesion growth or delayed visibility of initially ischemic tissue. This heterogeneity contributes to variability in reported incidence and clinical interpretation. Given the growing focus on tissue-level outcomes, a detailed characterization of new DWI lesions in the most favorable subgroup—patients achieving complete reperfusion—is needed. By restricting analysis to TICI 3 cases, confounding from residual macrovascular occlusion is minimized, allowing clearer assessment of microvascular failure and procedure-related embolization.

In this study, we evaluated the incidence, distribution, and morphological patterns of new DWI lesions in a homogeneous cohort of patients with anterior circulation LVO who achieved complete angiographic reperfusion. We also examined whether these lesions were associated with 90-day functional outcomes and mortality, with particular emphasis on clarifying their clinical relevance despite technically complete angiographic reperfusion. By focusing exclusively on patients with true TICI 3 reperfusions, our study aims to isolate tissue-level phenomena beyond angiographic resolution and contribute to the evolving concept of imaging-based definitions of reperfusion success.

## 2. Materials and Methods

### 2.1. Patient Selection and Study Design

This retrospective, single-center observational cohort study was conducted at a high-volume tertiary stroke center. We screened a consecutive database of 1313 patients who underwent MT for acute ischemic stroke between January 2022 and December 2024.

Eligibility criteria were:

1. Age ≥ 18 years;
2. Symptom onset to presentation ≤ 8 h;
3. Anterior circulation large-vessel occlusion involving the internal carotid artery terminus (ICA-T) or the middle cerebral artery (MCA) M1 segment;
4. Baseline imaging demonstrating salvageable brain tissue;
5. True complete reperfusion with no residual distal occlusion or branch cut-off on the final DSA run [25,26,27];
6. High-quality follow-up DWI performed within 48 h post-procedure.

Baseline imaging selection was performed using multimodal computed tomography (CT), including CT perfusion, with salvageable tissue defined as a mismatch between ischemic core and hypo perfused tissue according to institutional stroke protocol. Quantitative penumbra volumes were not consistently available due to the retrospective design.

**Figure 1:**
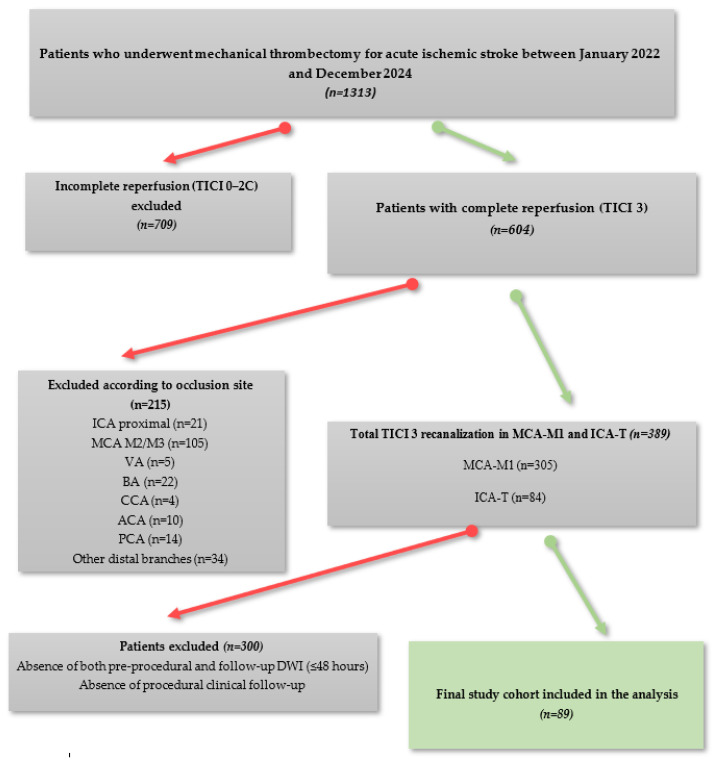
Flowchart of study group selection. Abbreviations: TICI, Thrombolysis in Cerebral Infarction; ICA, internal carotid artery; MCA, middle cerebral artery; VA, vertebral artery; BA, basilary artery; CCA, common carotid artery; ACA, anterior cerebral artery; PCA, posterior cerebral artery; DWI, diffusion-weighted imaging.

Patients with posterior circulation stroke, isolated distal occlusions (M2/M3), incomplete reperfusion (TICI < 3), or non-standardized pre- or post-procedure MRI were excluded. The final analysis included 89 patients (Figure 1). This cohort represents a highly selected subgroup of patients with both true TICI 3 reperfusion and standardized follow-up MRI, rather than the overall institutional reperfusion rate. Out of 1313 patients screened, only 89 met all inclusion criteria, reflecting the application of strict selection criteria, including confirmed true complete (TICI 3) reperfusion and availability of high-quality, standardized follow-up MRI within 48 h.

### 2.2. MRI Acquisition and Sequence Parameters

All MRI examinations were performed on a 1.5 T scanner (Philips Ingenia 1.5T; Philips Healthcare, Best, The Netherlands) with a 16-channel head-neck coil. The acute stroke protocol included axial DWI, fluid-attenuated inversion recovery (FLAIR), susceptibility-weighted imaging (SWI), and additional standard sequences as per institutional protocol.

Follow-up MRI was performed within 48 h after thrombectomy to capture early post-procedural ischemic changes while minimizing the influence of delayed infarct evolution. This time window is commonly used in prior studies to balance early lesion detection and imaging reliability [14,23].

DWI was acquired using single-shot spin-echo echo-planar imaging with the following parameters: repetition time (TR) 3400 ms; echo time (TE) 92 ms; matrix 152 × 122 (reconstructed to 256 × 256); field of view (FOV) 230 × 230 mm; slice thickness 5 mm with a 1 mm interslice gap; 24 axial slices; b-values of 0 and 1000 s/mm^2^; and diffusion encoding in three orthogonal directions. Apparent diffusion coefficient (ADC) maps were generated for all patients and were used to confirm true diffusion restriction and exclude T2 shine-through.

### 2.3. Radiological Image Analysis

New DWI lesions were defined as focal areas of diffusion restriction that were absent on baseline DWI and newly appeared on follow-up DWI within 48 h. All lesions were required to demonstrate corresponding low signals on ADC maps to confirm true diffusion restriction.

Paired comparison of baseline and follow-up DWI and ADC images was performed in all cases. Lesions were classified as “new” only if they were spatially distinct from the baseline infarct core and could not be explained by lesion growth or extension within the original vascular territory.

Two board-certified radiologists (each with >10 years of experience), blinded to clinical outcomes and procedural details, independently reviewed all imaging data. Discrepancies were resolved by consensus.

Lesions were categorized according to:

1. Laterality: ipsilateral, contralateral, or bilateral relative to the recanalized vessel;
2. Morphology: millimetric cortical foci (<5 mm), territorial infarcts (>1/3 of a vascular territory), or isolated basal ganglia infarcts;
3. Lobar distribution: frontal, temporal, parietal, or occipital.

Although SWI sequences were included in the imaging protocol, systematic evaluation of distal susceptibility signals corresponding to new DWI lesions was not performed.

### 2.4. Clinical Assessment and Functional Outcome

Neurological status was assessed using the National Institutes of Health Stroke Scale (NIHSS) at baseline, 24 h after the procedure, and at discharge. NIHSS scores are reported as median (interquartile range, IQR).

Stroke etiology was classified according to the TOAST (Trial of Org 10172 in Acute Stroke Treatment) criteria.

The primary clinical endpoint was the 90-day functional outcome, assessed using the modified Rankin Scale (mRS). To ensure consistency and reduce inter-observer variability, mRS scores were obtained using the Rankin Focused Assessment [28]. Assessments were performed by a certified stroke coordinator during outpatient visits or via structured telephone interviews when necessary. Favorable functional outcome was defined as mRS 0–2. Mortality was recorded as all-cause death within 90 days.

### 2.5. Statistical Analysis

Statistical analyses were performed using IBM SPSS Statistics, version 26.0 (IBM Corp., Armonk, NY, USA). Continuous variables are presented as mean ± standard deviation (SD) or IQR, as appropriate. Categorical variables are presented as counts and percentages.

Between-group comparisons (patients with new DWI lesions vs. those without) were performed using the independent-samples *t*-test or Mann–Whitney U test for continuous variables, and the chi-square or Fisher’s exact test for categorical variables, as appropriate. A two-sided *p*-value < 0.05 was considered statistically significant.

Multivariable analysis was not performed due to the limited sample size and number of outcome events.

### 2.6. Ethical Approval

This study was approved by the Institutional Review Board (approval no: 184/2025, date: 2 September 2025) and conducted in accordance with the Declaration of Helsinki. The requirement for informed consent was waived due to the retrospective design.

## 3. Results

### 3.1. Baseline and Procedural Characteristics

**Figure 2:**
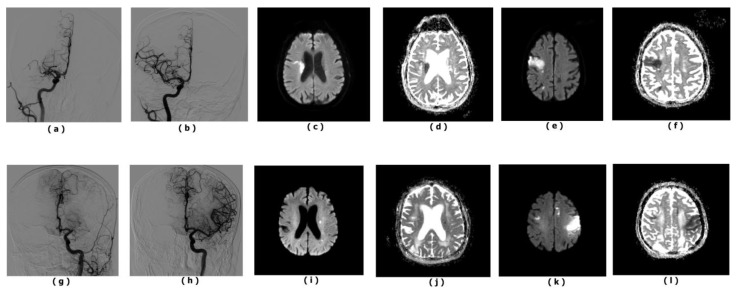
Representative cases of new DWI lesions after complete (TICI 3) reperfusion following mechanical thrombectomy. (**a**) Case 1 pre-thrombectomy digital subtraction angiography (DSA) demonstrating occlusion of the right middle cerebral artery (MCA) M1 segment. (**b**) Post-thrombectomy DSA showing complete reperfusion (TICI 3) with full restoration of distal flow. (**c**,**d**) Baseline diffusion-weighted imaging (DWI) and corresponding apparent diffusion coefficient (ADC) maps showing the initial infarct core. (**e**,**f**) Follow-up DWI and ADC images obtained within 48 h demonstrating newly developed ipsilateral frontal and parietal lobe cortical diffusion-restricted lesions, spatially distinct from the baseline infarct. (**g**) Case 2 pre-thrombectomy DSA demonstrating MCA M1 segment occlusion. (**h**) Post-thrombectomy DSA showing complete reperfusion (TICI 3). (**i**,**j**) Baseline DWI and ADC images showing small initial foci. (**k**,**l**) Follow-up DWI and ADC images demonstrating new diffusion-restricted lesions, including ipsilateral and contralateral foci. All new lesions were confirmed by corresponding low signal on ADC maps to exclude T2 shine-through. Follow-up MRI was performed within 48 h after the procedure.

The final study cohort consisted of 89 patients with a mean age of 69.4 ± 12.3 years, all of whom had anterior circulation large-vessel occlusion and achieved complete angiographic reperfusion. The primary occlusion sites were the MCA M1 segment in 67 patients (75.3%) and the ICA-T in 22 patients (24.7%) (Figure 2a–l).

Comparisons between patients with and without new DWI lesions showed no significant differences across baseline demographic, clinical, or procedural variables, including age (*p* = 0.308), sex (*p* = 0.764), and intravenous alteplase administration (*p* = 0.724). Procedural metrics, such as the number of thrombectomy passes (*p* = 0.306) and groin-to-recanalization time (*p* = 0.902), were also similar between groups (Table 1).

Additional baseline clinical characteristics, including vascular risk factors (hypertension, diabetes mellitus, atrial fibrillation, hyperlipidemia, and smoking status) and TOAST classification, were analyzed. No significant differences were observed between patients with and without new DWI lesions for any of these variables (all *p* > 0.05).

The distribution of occlusion sites (M1 vs. ICA-T) did not differ significantly between patients with and without new DWI lesions (*p* = 0.824).

### 3.2. Patterns of New DWI Lesions

New DWI lesions were detected on follow-up MRI in 28 of 89 patients (31.5%) within 48 h after MT. Among these patients, 22 (78.6%) had M1 occlusions and 6 (21.4%) had ICA-T occlusions.

Millimetric cortical foci were the predominant morphological pattern, observed in 24 of 28 patients (85.7%). Large territorial infarcts were identified in 4 patients (14.3%), and isolated basal ganglia infarcts in 4 patients (14.3%). Because multiple lesion types coexisted in some patients (n = 4), these categories were not mutually exclusive.

New lesions were predominantly ipsilateral to the recanalized vessel in 22 patients (78.6%). Contralateral lesions occurred in 4 patients (14.3%), while bilateral involvement was seen in 2 patients (7.1%). No significant association was found between occlusion site and lesion laterality (*p* = 0.250).

In M1 occlusions, lesions were most frequently located in the frontal (45.5%), temporal (36.4%), and parietal (31.8%) lobes. In ICA-T occlusions, lesions were most commonly observed in the parietal (50.0%) and frontal (33.3%) lobes. There was no statistically significant association between occlusion site and lobar distribution (*p* = 0.294) (Table 2).

### 3.3. Neurological and Functional Outcomes

NIHSS scores improved at 24 h compared to baseline in both groups; however, the magnitude of improvement did not differ significantly between patients with and without new DWI lesions (*p* = 0.638).

The presence of new DWI lesions was not associated with 90-day functional outcomes (*p* = 0.930) or 90-day survival (*p* = 0.613). Rates of favorable functional outcome (mRS 0–2 at 90 days) were comparable between patients with and without new DWI lesions (Table 3).

## 4. Discussion

The present study demonstrates that approximately one-third (31.5%) of patients with anterior circulation large-vessel occlusion who achieve TICI 3 develop new DWI-restricted lesions within 48 h of MT. This finding underscores a significant clinic–radiological dissociation: restoration of macrovascular patency—even to its most complete extent—does not inherently equate to full tissue-level protection. Despite the prevalence of these lesions, their presence was not associated with worse 90-day functional outcomes, suggesting that small-volume ischemic injuries may remain clinically silent in the setting of successful reperfusion.

### 4.1. The Incidence of New DWI Lesions: Beyond the Angiographic Eye

The incidence of 31.5% observed in our cohort falls within the broad range (10–50%) reported in previous studies evaluating post-thrombectomy imaging [13,14,15]. However, our study exclusively included TICI 3 patients, representing the most favorable procedural subgroup. In more heterogeneous populations—including those with TICI 2b or lower reperfusion grades—the incidence of new lesions is often higher due to residual distal occlusions or persistent hypoperfusion [7,8].

The emergence of these lesions after “complete” recanalization highlights the limitations of DSA in detecting microvascular failure. As Schönfeld et al. [13] demonstrated using high-resolution DWI, many of these lesions represent sub-angiographic peripheral emboli—fragments too small to be visualized on standard procedural imaging yet sufficient to induce focal cytotoxic edema detectable on MRI. In our cohort, all lesions were confirmed by corresponding ADC reduction, supporting true diffusion restriction rather than imaging artifacts.

Recent meta-analytic evidence further emphasizes that radiological and clinical dissociation after thrombectomy remains a persistent challenge even in technically successful procedures. Kiani et al. [29] demonstrated that imaging-based predictors of futile recanalization extend beyond angiographic scores and include tissue-level markers of microvascular compromise. Similarly, Umemura et al. [30] highlighted that infarct core characteristics and post-reperfusion tissue dynamics may influence outcomes despite apparent macrovascular success. Our findings align with this evolving concept, reinforcing that TICI 3 reperfusion does not necessarily equate to complete microvascular restoration.

### 4.2. Mechanisms of New Lesion Formation: Embolic Shower and Microvascular Integrity

The anatomical and morphological patterns identified in our study provide insight into the underlying pathophysiology. The predominance of ipsilateral (78.6%) and millimetric cortical foci (85.7%) strongly suggests a procedure-related embolic mechanism. During MT, thrombus manipulation by stent retrievers or aspiration catheters may result in clot fragmentation, propelling small emboli distally into the microcirculation [12,18]. Although technical refinements such as balloon guide catheters or protected retrieval techniques aim to reduce distal embolization [18], complete elimination of micro embolic phenomena remains challenging.

Beyond mechanical fragmentation, the no-reflow phenomenon likely contributes to tissue-level injury. As reviewed by Sperring et al. [10], distal microvascular obstruction may persist despite large-vessel patency due to pericyte-mediated capillary constriction, endothelial swelling, and thrombo-inflammatory processes [12]. This microvascular dysfunction can prevent restoration of effective nutritive blood flow, manifesting as new DWI-restricted lesions.

The presence of contralateral (14.3%) and bilateral (7.1%) lesions, although less frequent, suggests that embolic material may occasionally traverse collateral pathways such as the circle of Willis or originate from proximal sources during catheter manipulation [25].

Time metrics also deserve consideration. Although we did not observe a significant association between groin-to-recanalization time and new lesion formation, procedural duration has been identified as an important determinant of outcome in broader stroke populations. De Mase et al. [31] demonstrated that groin-to-recanalization time remains a major predictor of clinical recovery, underscoring that even brief periods of microvascular ischemia may have downstream effects. Our findings suggest that while procedural speed is critical for overall outcome, micro embolic phenomena may occur independently of time efficiency in cases of complete angiographic reperfusion.

### 4.3. The Clinical Impact of “Silent” Infarcts

A key finding of our study is the lack of association between new DWI lesions and 90-day functional outcomes. This aligns with prior reports suggesting that small-volume, predominantly cortical lesions may be functionally compensated, particularly when surrounding penumbral tissue has been successfully salvaged following TICI 3 reperfusions [23,24].

However, caution is warranted regarding the term “silent.” While these lesions may not significantly influence global disability scales such as the mRS, accumulating evidence suggests potential long-term consequences. Vermeer et al. [20] and Debette et al. [21] demonstrated that silent brain infarcts are associated with increased risks of cognitive decline and dementia, while Wardlaw et al. [22] emphasized the cumulative burden of small-vessel injury over time. Therefore, although our findings provide reassurance regarding short-term functional outcomes, the long-term clinical significance of these lesions warrants further investigation.

### 4.4. The Evolution of Reperfusion Grading: From TICI to eTICI

Historically, TICI 2b (50–99% filling) was considered the threshold for procedural success. However, angiographic grading systems were originally designed to reflect macrovascular reperfusion rather than tissue-level restoration [32]. Subsequent analyses have demonstrated that TICI 3 reperfusion is associated with superior outcomes compared to TICI 2b [7,26], prompting the development of expanded grading systems such as e-TICI [25,27].

Our findings suggest that even within the TICI 3 subgroup, a subset of patients experiences ongoing tissue injury detectable only on diffusion imaging. This observation supports the integration of imaging-based endpoints into future reperfusion paradigms and highlights the need to move beyond purely angiographic definitions of procedural success.

### 4.5. Limitations and Future Directions

Despite the strengths of a homogeneous TICI 3 cohort and standardized imaging protocol, several limitations should be acknowledged. The retrospective design introduces potential selection bias. The sample size, while sufficient to identify common patterns, may be underpowered to detect subtle clinical differences. Additionally, volumetric quantification of new lesions was not performed; however, morphological classification served as a pragmatic surrogate for lesion burden.

Importantly, the strict inclusion criteria resulted in a highly selected cohort, with only 89 out of 1313 screened patients meeting all eligibility criteria. While this approach strengthens the internal validity of the study by minimizing confounding factors such as incomplete reperfusion or non-standardized imaging, it may limit the generalizability of the findings to broader stroke populations.

Future studies incorporating automated volumetric analysis, multimodal perfusion imaging, and long-term cognitive follow-up will be essential to further elucidate the clinical trajectory of patients with post-thrombectomy DWI lesions.

## 5. Conclusions

In this strictly defined cohort of patients achieving true complete angiographic reperfusion, new DWI lesions were observed in approximately one-third of cases following mechanical thrombectomy. These findings reinforce accumulating evidence that complete angiographic reperfusion does not necessarily translate into full tissue-level protection.

Importantly, the predominantly millimetric, cortical, and largely ipsilateral nature of these lesions may explain why they were not associated with worse 90-day functional outcomes. In the setting of successful reperfusion, preservation of surrounding penumbral tissue likely mitigates their clinical impact, rendering many of these lesions functionally silent on global disability measures such as the modified Rankin Scale.

Nevertheless, their presence reflects ongoing microvascular or embolic injury beyond the resolution of angiographic assessment, consistent with prior studies highlighting the limitations of angiography in capturing tissue-level reperfusion. Overall, these findings support the need for integrated, tissue-level definitions of reperfusion success and emphasize the need for strategies aimed at minimizing distal embolization and preserving microvascular integrity.

## Figures and Tables

**Table 1:** Baseline and Procedural Characteristics of Patients with and without New DWI Lesions.

Variable	New DWI (−) (*n* = 61)	New DWI (+) (*n* = 28)	*p*-Value
Age, years *	68.44 ± 12.87	71.32 ± 10.92	0.308
Sex, *n* (%)			
Female	32 (52.5)	13 (46.4)	0.764
Male	29 (47.5)	15 (53.6)	
Intravenous alteplase, *n* (%)			
Yes	5 (8.2)	1 (3.6)	0.724
No	56 (91.8)	27 (96.4)	
Number of thrombectomy passes *	1.44 ± 0.70	1.29 ± 0.60	0.306
Groin-to-recanalization time, min *	32.56 ± 12.41	32.89 ± 10.67	0.902
Hypertension, *n* (%)	35 (57.4)	20 (71.4)	0.368
Diabetes mellitus, *n* (%)	23 (37.7)	9 (32.1)	0.650
Atrial fibrillation, *n* (%)	24 (39.3)	8 (28.6)	0.511
Hyperlipidemia, *n* (%)	7 (11.5)	5 (17.9)	0.511
Smoking, *n* (%)	11 (18.0)	6 (21.4)	0.768
TOAST classification, *n* (%)			
Large-artery atherosclerosis (Type 1)	25 (41.0)	11 (39.3)	0.804
Cardio embolism (Type 2)	20 (32.8)	8 (28.6)	
Small-vessel occlusion (Type 3)	0 (0.0)	0 (0.0)	
Other determined etiology (Type 4)	1 (1.6)	0 (0.0)	
Undetermined etiology (Type 5)	15 (24.6)	9 (32.1)	

* Data are presented as mean ± standard deviation (SD). Categorical variables are presented as *n* (%). Abbreviations: DWI, diffusion-weighted imaging; TOAST, Trial of Org 10172 in Acute Stroke Treatment.

**Table 2:** Morphological Characteristics and Distribution of New DWI Lesions (*n* = 28).

Variable	*n* (%)
Morphology	
Millimetric cortical foci	24 (85.7)
Large territorial infarcts	4 (14.3)
Isolated basal ganglia infarcts	4 (14.3)
Laterality	
Ipsilateral	22 (78.6)
Contralateral	4 (14.3)
Bilateral	2 (7.1)

Abbreviations: DWI, diffusion-weighted imaging. Categories are not mutually exclusive; some patients had more than one lesion type.

**Table 3:** Neurological and Functional Outcomes.

Variable	New DWI (−) (*n* = 61)	New DWI (+) (*n* = 28)	*p*-Value
NIHSS score at admission *	18 (16–20)	19 (15–22)	0.376
NIHSS score at 24 h *	6 (2–10)	5 (3–13.3)	0.638
90-day modified Rankin Scale *	1 (1–3)	1 (1–2.3)	0.930
90-day survival, *n* (%)			
Alive	56 (91.8)	24 (85.7)	0.613
Deceased	5 (8.2)	4 (14.3)	

* Data are presented as median (interquartile range, IQR). Categorical variables are presented as n (%). Abbreviations: DWI, diffusion-weighted imaging; NIHSS, National Institutes of Health Stroke Scale.

## Data Availability

The datasets generated and/or analyzed during the current study are not publicly available due to institutional data protection policies but are available from the corresponding author on reasonable request.

## Abbreviations

The following abbreviations are used in this manuscript:

DWI	Diffusion-Weighted Imaging
MRI	Magnetic Resonance Imaging
MT	Mechanical thrombectomy
TICI	Thrombolysis in Cerebral Infarction
mRS	Modified Rankin Scale
NIHSS	National Institutes of Health Stroke Scale
TOAST	Trial of Org 10172 in Acute Stroke Treatment
MCA	Middle Cerebral Artery
ICA	Internal Carotid Artery
LVO	Large-Vessel Occlusion
DSA	Digital Substruction Angiography
SPSS	Statistical Package for the Social Sciences
